# Investigating knowledge regarding antibiotics and antimicrobial resistance among pharmacy students in Sri Lankan universities

**DOI:** 10.1186/s12879-018-3107-8

**Published:** 2018-05-08

**Authors:** M. H. F. Sakeena, Alexandra A. Bennett, Shazia Jamshed, Fahim Mohamed, Dilanthi R. Herath, Indika Gawarammana, Andrew J. McLachlan

**Affiliations:** 1 Department of Pharmacy, Faculty of Allied Health Sciences, University of Peradeniya, Peradeniya, Sri Lanka; 2 Sydney Pharmacy School, The University of Sydney, Sydney, NSW Australia; 3 NSW Therapeutic Advisory Group, Darlinghurst, NSW Australia; 4 Department of Pharmacy Practice, Kulliyyah of Pharmacy, International Islamic University Malaysia, Kuantan, Pahang Malaysia; 5 Discipline of Pharmacology, Sydney Medical School, University of Sydney, Sydney, NSW Australia; 6 Department of Medicine, Faculty of Medicine, University of Peradeniya, Peradeniya, Sri Lanka; 7 Centre for Education and Research on Ageing, Concord Repatriation General Hospital, Sydney, NSW Australia

**Keywords:** Antibiotics, Antimicrobial resistance, Pharmacy education, Knowledge, Sri Lanka, South Asia, Developing countries

## Abstract

**Background:**

Antimicrobial resistance (AMR) is a major challenge for global health care. Pharmacists play a key role in the health care setting to help support the quality use of medicines. The education, training, and experiences of pharmacy students have the potential to impact on patterns of antibiotic use in community and hospital settings. The aim of this study was to investigate antibiotic use, knowledge of antibiotics and AMR among undergraduate pharmacy students at Sri Lankan universities and to compare this between junior and senior pharmacy student groups.

**Methods:**

A cross-sectional study was conducted at the six universities in Sri Lanka that offer pharmacy undergraduate programmes. All pharmacy students in each university were invited to participate in this study using a self-administered questionnaire with ethics approval. The study instrument comprised five major sections: demographic information, self-reported antibiotic use, knowledge of antibiotic uses in human health, knowledge of AMR and antibiotic use in agriculture. Descriptive data analyses were conducted and Chi-squared analysis was used to explore associations between different variables and level of pharmacy education.

**Results:**

Four hundred sixty-six pharmacy students completed the questionnaire. A majority of participants (76%) reported antibiotic use in the past year. More than half (57%) of the junior pharmacy students incorrectly indicated that antibiotic use is appropriate for the management of cold and flu conditions. Senior pharmacy students (*n* = 206) reported significantly better antibiotic knowledge than junior students (*n* = 260), *p* < 0.05. Overall pharmacy students showed good understanding of AMR and their knowledge level increased as the year of pharmacy study increased.

**Conclusions:**

This study found that pharmacy students commonly report using antibiotics. Junior students report some misconceptions about antimicrobials. A comparison between junior and senior pharmacy students suggests that pharmacy education is associated with improved understanding of appropriate antibiotic use and AMR among undergraduate pharmacy students in Sri Lanka.

**Electronic supplementary material:**

The online version of this article (10.1186/s12879-018-3107-8) contains supplementary material, which is available to authorized users.

## Background

The increase of resistance to commonly used antimicrobial agents has become a major challenge for public health [1]. Misuse and overuse of antimicrobial agents are the major factors contributing to the development and spread of antimicrobial resistance (AMR) worldwide [2]. According to the World Health Organization (WHO), the world is heading towards a ‘post-antibiotic era.’ It is predicted that common infections and minor health injuries will become a key threat to public health and the WHO has urged countries to make immediate resolutions to overcome this global issue [3]. The populations of low and middle income countries are considered to be at greater public health risk given the relatively high prevalence of infection, over-the-counter availability of antibiotics, the lack of therapeutic guidelines for prescribers and limited access to microbiological testing to identify infections quickly and cost effectively [4].

In this context, the role of health care workers knowledgeable in the appropriate use of antibiotics and prevention of AMR is critical to the global efforts to reduce AMR and sustain antibiotic effectiveness. An essential component of the WHO global action plan is a need to improve awareness and understanding of antibiotics and AMR through effective communication, education and training to control further expansion of AMR [3]. Several studies have highlighted that education about antibiotics and their appropriate use during undergraduate training has significant impact on a professional’s attitudes and behaviour regarding antibiotic use [5, 6]. This training is critical for students of professional health care degrees, such as medicine, pharmacy and nursing.

Pharmacists are an important member in the healthcare team and have a major role in medicine use and the provision of advice regarding appropriate medicines use and health care [7, 8]. Pharmacist education and training have the potential to influence the behaviour of other health professionals and consumers [9, 10] as a multidimensional strategy for changing practice to ensure the quality use of medicines [11]. Pharmacists are well placed to improve the understanding of antibiotics and their judicious use by direct contact with the consumers in the community [12] and in hospital [13] and raising awareness of consumers regarding appropriate antibiotic use [14]. Inadequate education and inappropriate training of pharmacist in some countries, particularly developing countries, can contribute to substandard professional practices [15, 16] which may lead to pharmacists having a tendency to inappropriate use, recommend and supply antibiotics [17] including the non-prescription sales of antibiotics in community pharmacies [18]. Pharmacists are the gatekeepers of appropriate medicine use including antibiotics. Comprehensive and relevant education and training on the use of antibiotics and AMR is essential for pharmacists to take a leading role in changing behaviours around antibiotic consumption and the appropriate use of antimicrobial agents in the community.

Sri Lanka is a developing country in South Asia with well-established legislation regulating the supply of antibiotics [19]. Antibiotic should only be dispensed on an authentic prescription from an appropriate prescriber, typically a doctor [19]. In Sri Lanka therapeutic guidelines are also available for prescribers [20] which emphasize evidence-based empirical and prophylactic prescribing of antibiotics [20]. Currently, little is known about how pharmacy students in low-income countries such as Sri Lanka perceive antibiotic usage and AMR. An essential aspect of this research is the focus on understanding antibiotic use among young adults with an educational background in health science. Moreover, interventions and strategies that will impact the emergence and growth of AMR in Sri Lanka will need to be designed based on information derived from Sri Lanka and relevant to the Sri Lankan health care system.

Therefore, the aims of this study are to investigate pharmacy students’ antibiotic use, knowledge about antibiotics and AMR and to evaluate difference in these outcomes between junior and senior pharmacy students.

## Methods

### Study design and setting:

This study was a descriptive cross-sectional study investigating the prevalence of antibiotic use, knowledge of antibiotics and knowledge of antimicrobial resistance (AMR) among undergraduate pharmacy students in universities in Sri Lanka. The study was conducted in the six universities of Sri Lanka (from different geographical region) where Bachelor of Pharmacy (B.Pharm) programmes are offered. This includes four state universities: University of Peradeniya (UoP), University of Sri Jayewardenepura (USJP), University of Jaffna (UoJ), University of Ruhuna (UoR); one military academy: Kothalawala Defence University (KDU); and, one public university with distance mode of learning: Open University of Sri Lanka (OUSL).

### Sample size

The sample size for this study was calculated based on achieving a representative sample of the total number of pharmacy students enrolled in undergraduate pharmacy (B. Pharm) programmes in each university in Sri Lanka. The study was able to cover above 80% of response rate from five universities in Sri Lanka. Total numbers of students enrolled at each university across all years were 114 pharmacy students at UoP, 111 at USJP, 78 at UoJ, 75 at UoR, 103 at KDU and 257 at OUSL. Pharmacy undergraduate degrees are a four-year study programme in Sri Lanka. The total number of pharmacy students in each Sri Lankan university is relatively low (20 to 30 students in each year cohort). The total number of students enrolled in pharmacy undergraduate degree program in each university during the data collection is listed in Additional file 1: Annexure 1. In this study, students in the first 2 years of the degree were considered “junior” pharmacy students and students in the later years, “senior” students.

### Inclusion and exclusion criteria

All male and female students enrolled in B.Pharm programmes at the universities of Sri Lanka during the study period were eligible to take part in the study. Students who were not willing to take part in this survey voluntarily were excluded from the study.

### Survey administration and data collection procedure:

Approval to collect the data from pharmacy undergraduate students was obtained before data collection from senior officials at relevant universities. Permission to meet the students in their respective universities was obtained from coordinators of B.Pharm programmes. The lead researcher visited the six universities to explain and administer the survey and collect data. Students were formally invited into a classroom to participate in the study. A self – administered questionnaire was distributed following a short introduction to the research project and written informed consent was obtained from the study participants. Completed self-administered questionnaires were returned to the researcher on the same day. All surveys were completed between January and April 2016.

### Data collection tool

A paper-based questionnaire was used for the data collection. The questionnaire used in this study is based on the World Health Organization (WHO): Antibiotic Resistance, Multi-country public awareness survey [21], used by the authors [22] and other researchers [23, 24]. The complete WHO questionnaire was used in this study (Additional file 2). This questionnaire was selected because of its previous use by WHO and its comprehensive nature that included relevant topics of antibiotics use, knowledge on antibiotics and knowledge on antimicrobial resistance. Permission was obtained from WHO to reprint and reproduce the survey (WHO reference number 239656). The content and format of the questionnaire used in this study were evaluated for face validity using a pre-test involving 10 B. Pharm students from University of Peradeniya. In the pre-test, pharmacy students provided feedback on the design, relevance, readability, the terminology used, and the flow of individual questions between sections of the questionnaire. The face validity of the questionnaire was also assessed by a senior pharmacist from NSW Therapeutic Advisory Group, NSW, Australia. Importantly, this questionnaire has been successfully tested in 9772 participants from 12 different countries [21] by WHO as part of the implementation objective of one of the global action plan on AMR and employed among 666 young university students and 131 seniors attending courses at the university in Italy [24] from schools of medical, dental, nursing, health promotion and dietetics [24].

The questionnaire was presented in English, which is the language of instruction in Sri Lankan universities. The questionnaire consisted of five major sections (A, B, C, D and E) and all were close ended questions as outlined below. A: Basic demographic information: including age, gender, name of the university and year of university study. B: Antibiotic Use: 4 questions about the past use of antibiotics, information about the purchase of antibiotics with a prescription or by self-prescribing, advice obtained at the time of purchase from the health care professional and the place where antibiotics were obtained. C: Knowledge of antibiotics: 4 questions about the duration of treatment, knowledge on sharing antibiotics, symptoms justifying use of antibiotics and knowledge on the use of antibiotics for appropriate disease conditions. D: Knowledge of antibiotic resistance: 5 questions about AMR and commonly used terms related to AMR; eight True/ False statements on antibiotic resistance and eight statements regarding AMR on a five-point “Likert scale”. E: Antibiotics in the community: one question on antibiotic use in agriculture and food products. Internal consistency of survey questions was validated using Cronbach’s alpha. The criterion for accepting Cronbach’s alpha for this study was a score above 0.7.

### Data analysis:

Data was entered into Statistical Process for Social Sciences (SPSS) software (Version 22.0 SPSS IBM, USA). Descriptive data analyses were undertaken including frequencies and percentages. Chi-squared analysis was used to test for significant associations between variables and level of pharmacy education (junior vs senior). The limit for statistically significant differences was set at *p <* 0.05.

### Ethical considerations

Approval letters were sought and obtained from the Head or Coordinator of each of the pharmacy degree programs from Sri Lankan universities participating in this research study. These approval letters were submitted, together with the ethics application, to the human ethics research committee at the University of Peradeniya. This institutional ethical clearance committee was able to provide ethical approval (Reference number 2015/EC/82) for the conduct of this study at each of the participating universities. All six universities accepted the ethical approval from the institutional ethical clearance committee, Faculty of Medicine, University of Peradeniya, Sri Lanka. The participants were assured of the confidentiality and anonymity of the information they provided. No financial inducements were offered to participants.

## Results

### Demographic data

**Fig. 1:**
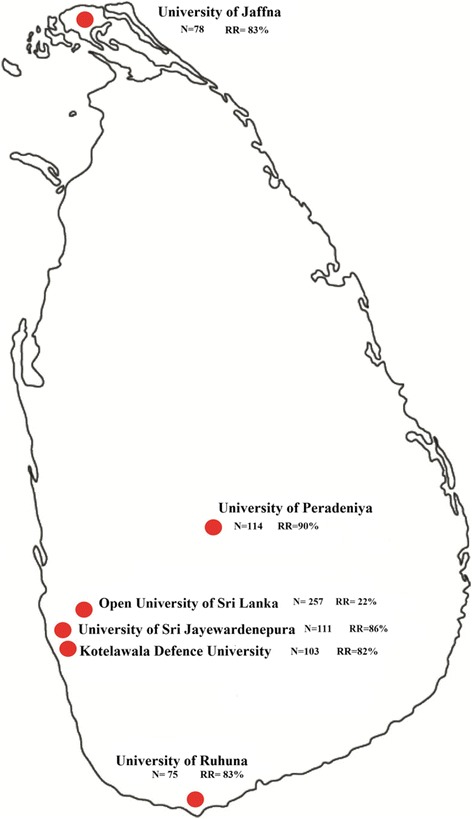
Eligible undergraduate pharmacy students at Sri Lankan universities and study response rates. *N* = number of students enrolled for pharmacy programme during data collection. RR = Response rate

The demographics of study participants and the response rate of each university are summarized in Table 1 and Fig. 1. A total of 466 B.Pharm students completed the study with a response rate above 80% for each university except the university which provides distance mode of learning (Additional file 1). The majority of the students (76%) were aged between 20 and 25 years and were predominately female (67%). Good representation was obtained across the four years of study; the proportions of first, second, third and fourth year of pharmacy students were 30%, 26%, 25% and 19%, respectively.

**Table 1:** Characteristics of study participants (*n* = 466)

Variable	Junior Students (*n* = 260)	Senior Students (*n* = 206)
	Frequency (%)	Frequency (%)
Age		
20–25	220 (85)	136 (66)
26–34	21 (8)	53 (26)
35–44	16 (6)	10 (5)
45+	3 (1)	7 (3)
Gender		
Male	80 (31)	71 (34)
Female	179 (69)	135 (66)
University		
UoP	59 (23)	43 (21)
USJP	48 (18)	48 (23)
UoJ	44 (17)	21 (10)
UoR	35 (13)	27 (13)
KDU	44 (17)	40 (19)
OUSL	30 (12)	27 (13)
Year of Study		
1st	139 (53)	0
2nd	121 (47)	0
3rd	0	116 (56)
4th	0	90 (44)

University of Peradeniya (UOP), University of Sri Jayewardenepura (USJP), University of Jaffna (UOJ), University of Ruhuna (UOR), Kothalawala Defence University (KDU), Open University of Sri Lanka (OUSL)

### Antibiotic use

The frequency of the use of antibiotics for pharmacy students in Sri Lankan universities during the last year was high (75%) with a majority (77%) of the students reporting that they have purchased antibiotics by prescription. Over half the pharmacy students (57%) reported that they received advice from a health care professional when purchasing antibiotics about how to take the antibiotics. However a sizeable proportion did not receive advice (35%) or could not remember obtaining advice (6%). The majority of students (90%) purchased antibiotics from a pharmacy rather than non-pharmacy suppliers such as stalls, hawkers, the internet, friends and family members.

### Comparison of antibiotic use between junior and senior pharmacy students

Past personal use of antibiotics did not differ significantly in either the last month, last six months or the last year among junior pharmacy students (*n* = 260) and senior students (*n* = 206). For instance, 32% of junior pharmacy students and 24% of senior students reported that they had used antibiotics in the last month. A majority of junior pharmacy students (79%) and senior students (78%) reported that they purchased antibiotics on prescription while 19% of junior students and 20% of senior students reported they purchased without a prescription. It is noteworthy that 50% of the senior pharmacy students were confident about their antibiotic use and did not report receiving advice from doctors, nurses, and pharmacist. In contrast, the majority of the junior students (69%) reported that they depended on the advice of health care professionals regarding antibiotic use. Pharmacy students reported mixed views on the duration of antibiotic use with 60% of junior pharmacy students and 87% of senior students believing antibiotics should be taken until the full course was completed while 30% of junior pharmacy students and 10% of senior students reported that the duration of antibiotic treatment should be limited and ceased when a person feels better (Additional files 3 and 4).

### Knowledge on antibiotics

The proportion of pharmacy students reporting that antibiotics can be used to treat the following disease conditions were as follows: skin infections (82%), urinary tract infections (76%), sore throats (57%), cold and flu (51%), diarrhoea (49%) and fever (41%). It is interesting to note that some pharmacy students also reported that malaria (22%), HIV infection (11%), body aches (11%) and headaches (6%) can be treated by antibiotics.

### Comparison of knowledge on antibiotics between junior and senior pharmacy students

**Fig. 2:**
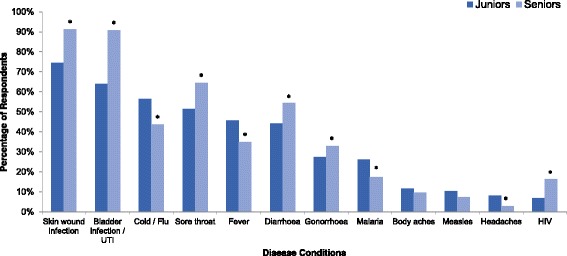
Percentages of pharmacy student respondents who reported they would use antibiotics in the listed disease conditions. HIV: Human Immunodeficiency Virus UTI**:** Urinary tract infection; Asterisk denotes *p* < 0.05, in comparison of senior (3rd and 4th years) and junior pharmacy students (1st and 2nd years

Figure 2 demonstrates that there were significant differences between junior pharmacy students and senior students in their knowledge regarding antibiotics and the disease conditions they should be used to treat. Junior pharmacy students scored poorly in their knowledge regarding the use of antibiotics with when to use antibiotics (Additional file 5). For example, a lower proportion of junior students (75%) correctly identified use of antibiotics for wound infections compared to the proportion of senior students (91%), *p <* 0.001. Moreover, 8% of junior students reported that they would frequently use antibiotics for headaches compared to 3% of the senior students, *p =* 0.02. Similarly, more junior pharmacy students (57%) would advocate use of antibiotics for symptoms of the common cold and flu than senior students (44%) *p* = 0.01.

### Knowledge of antibiotic resistance terminology

This study examined the source of students’ understanding of terminology related to antibiotic resistance as shown in Table 2. A large proportion of the total study population were familiar with the following terms: Antibiotic Resistance (94%), Antimicrobial Resistance (76%), Antibiotic Resistance Bacteria (88%) and Drug Resistance (80%). However, the terms ‘superbugs’ and ‘AMR’ were not well known among the pharmacy students (10% and 12% respectively). When asked about how they had become familiar with these terms, undergraduate studies (50%) followed by media (14%), pharmacists (10%), family member / friend (8%), doctor/ nurse (8%) and specific campaigns (4%) were the most common means (Additional file 6).

**Table 2:** Knowledge on terms related to antimicrobial resistance among undergraduate pharmacy students in the universities of Sri Lanka

Terms	Juniors (*n* = 260)	Seniors (*n* = 206)	*P* - value
	Frequency (%)	Frequency (%)	
Antibiotic Resistance	234 (90)	205 (100)	0.00*
Superbugs	28 (11)	18 (9)	0.465
Antimicrobial Resistance	168 (65)	186 (90)	0.00*
AMR	26 (10)	30 (15)	0.132
Drug Resistance	181 (70)	192 (93)	0.00*
Antibiotic Resistant Bacteria	210 (81)	198 (96)	0.00*

*Statistically significant

### Knowledge on antibiotic resistance

This study revealed that the students were aware of many key issues about antimicrobial resistance as shown in Table 3 and there were differences between junior pharmacy students’ and senior students’ knowledge of antibiotic resistance. Junior pharmacy students scored poorly in their knowledge regarding antimicrobial resistance. For instance, 80% of senior pharmacy students and 57% of junior students agreed that *“Many infections are becoming increasingly resistant to treatment by antibiotics”*. Further, 91% of senior pharmacy students and 75% of junior students disagreed with the following statement “*antibiotic resistance is an issue in other countries but not her*e”. The results suggest a good understanding of AMR among senior pharmacy students in Sri Lanka but significant gaps in AMR understanding among junior pharmacy students.

**Table 3:** Respondent numbers to various statements on antimicrobial resistance

Statements	Junior (*n* = 260)		Senior (*n* = 206)	
	Frequency	Percentage	Frequency	Percentage
ABR occurs when your body becomes resistant to AB and they no longer works as well				
True	234	90	205	99.5
False	26	10	1	.5
Don’t know	0	0	0	0
Many infections are becoming increasingly resistant to treatment by antibiotics				
True	149	57	164	80
False	56	22	27	13
Don’t know	47	18	13	6
If bacteria are resistant to AB, it can be very difficult or impossible to treat the infections they cause				
True	196	75	178	86
False	43	16	22	11
Don’t know	16	6	4	2
Antibiotic resistance is an issue that could affect me or my family				
True	165	64	170	82
False	42	16	19	9
Don’t know	43	16	13	6
ABR is an issue in other countries but not here				
True	8	3	3	2
False	196	75	188	91
Don’t know	51	20	13	6
ABR only a problem for people who take AB regularly				
True	63	24	36	18
False	167	64	159	77
Don’t know	24	9	10	5
Bacteria which are resistant to AB can spread from person to person				
True	147	56	129	63
False	63	24	49	24
Don’t know	44	17	25	12
ABR infections could make medical procedures, organ transplants and cancer treatment difficult				
True	150	58	154	75
False	27	10	9	4
Don’t know	79	30	42	20

*AB*: antibiotics, *ABR* antibiotic resistance

### Antibiotic use in agriculture and food producing animals

Less than half the pharmacy students (38%) reported that they knew about antibiotics usage in agriculture and food products when asked about the broader use of antibiotics in the community; 16% stated that antibiotics are not used in agriculture and 43% had no knowledge of antibiotics use in agriculture (Additional file 7).

## Discussion

### Statement of principal findings

The results of this study indicate a high degree of antibiotic use by undergraduate pharmacy students in the past year in Sri Lanka. Pharmacy students also reported some misconceptions about the appropriate use of antibiotics for conditions such cold and flu. Senior pharmacy students demonstrated significantly better understanding of the appropriate use of antibiotics when compared to their junior colleagues. Undergraduate education is likely to be the main source of improved knowledge of antibiotics, AMR, and related terminology. B.Pharm subjects such as pharmacology, clinical pharmacy, microbiology, and pathology courses offer the opportunity for antibiotic and AMR education. The curricula for pharmacy degree programs from different universities was not available to be assessed. However, the aim of this study was to investigate pharmacy students’ antibiotic use, knowledge about antibiotics and AMR including the secondary aim to evaluate possible differences in these outcomes between junior and senior pharmacy student groups. The results of this study identified that senior pharmacy students have significantly better knowledge (related to antibiotics and AMR) when compared to junior pharmacy students. Majority of these students answered ‘undergraduate studies’ as a source of their improved knowledge and understanding related to these concepts. Therefore, undergraduate education is likely to be the main source of improved knowledge of antibiotics, AMR, and related terminology among senior students.

### Context of these findings

Sri Lanka is one of the developing countries in South Asia. Sri Lanka has well established legislation regarding supply of antibiotics [19] and therapeutic guidelines are also available for prescribing [20]. However, pharmacy practice and clinical pharmacy remain emerging branches of pharmacy education in Sri Lanka. This current research is pertinent because it highlights that pharmacy education is associated with improved understanding of the appropriate use of antibiotics, and knowledge related to antibiotics and AMR among Bachelor of Pharmacy students in Sri Lanka. Targeted pharmacy education is likely to have an important role to play in the reduction of injudicious and inappropriate use of antibiotics. Sri Lanka has similar socio-economic status and challenges to many other developing countries. There are numerous factors that influence the practice of a pharmacist in a developing country like Sri Lanka including poorly monitored medicines regulation [25, 26], commercial pressure on pharmacy staff [27], consumer demand [28] and inappropriate prescribing practices [29]. These circumstances have the potential to lead to inappropriate practices of pharmacists. Therefore, multi-faceted strategies are essential to improve the appropriate use of antibiotics in Sri Lanka. In addition, collaborative approach to effective and strategic pharmacy education within Sri Lanka and South Asia is likely to reap significant rewards in the fight against AMR and the long term public health of their populations.

In Sri Lanka, there is currently no harmonised nationally implemented curriculum for undergraduate pharmacy (B.Pharm) programs. The typical undergraduate pharmacy curriculum involves studying basic and clinical science foundation modules taught in the early years of pharmacy undergraduate degree and pharmaco-therapeutics, clinical pharmacy courses are taught in later years. For example, at the University of Peradeniya, laboratory tests are introduced in microbiology in the pharmacy first year, pharmacology of antibiotics are taught in third year of study, and practice related concepts and therapeutics are taught in clinical pharmacy modules in their final year of study. While the details of specific curricula are not available to the authors this is the typical learning format.

### Strengths and weaknesses of the study

This study has investigated antibiotic use, knowledge related to antibiotics and AMR in a large and representative sample of pharmacy students in a South Asian country, Sri Lanka. The response rate of this nationwide survey of pharmacy students from six universities reflects the views of the majority of Sri Lankan undergraduate pharmacy students.

However, there were a few limitations in our study. We were not able to specifically link pharmacy undergraduate course content with students’ knowledge on antibiotics and AMR. Additionally, although this study used anonymous responses, we might expect that some students provided an answer that they believed the researcher would like to receive, especially about whether antibiotics were purchased on prescription or without a prescription.

### Strengths and weaknesses in relation to other studies, particularly related to differences in results

In this study the majority of the pharmacy students (92% junior, 93% senior students) reported they obtained antibiotics dispensed on a doctor’s prescription. These data reveal, students are reporting that they are adhering to the relevant legislation of Sri Lanka for access to antibiotics. This suggests that student’s have an appropriate attitude to the supply of antibiotics in the community. Earlier studies have found that university students in the field of medical and health sciences have the tendency to report self-prescribing practices due to the education and training they acquire during university [30–33]. However, in this study, greater number of pharmacy students reported obtaining antibiotics with an authentic prescription. Therefore, non-prescription use of antibiotics by Sri Lankan pharmacy students, was lower than that already identified in other studies from South Asia [30, 31]. Further, online pharmacies and websites selling prescription-only drugs including antibiotics is a challenge for many countries [34]. However, in this study no students reported purchasing antibiotics online. It is pleasing to note that the university student population in Sri Lanka do not have a practice of buying antibiotics through non-pharmacy suppliers such as stalls, hawkers, the internet, friends or family members.

There is an ongoing debate about the appropriate duration of antibiotic use. A number of recent articles have made controversial statements regarding the appropriate time to cease antibiotic therapy [35–37]. However, clinical guidelines recommend that antibiotic dose should be completed and taken as prescribed [38]. In this study, a sizeable proportion of senior pharmacy students reported that antibiotic course should be in accordance with the prescribed duration. On the other hand, a significant number of junior students reported that antibiotics should be stopped once the user feels better. It is interesting to note that pharmacy education appears to have made a considerable contribution to the understanding regarding the appropriate duration of antibiotic therapy.

Studies have found that symptoms related to respiratory tract infections such as cold and flu are frequent reasons for seeking health care services [39]. Other studies have suggested that university students widely use antibiotics for sore throat and cold and flu [31], resulting in inappropriate antibiotic use [40]. This study provides empirical support to the above-mentioned findings with a significant number of junior students apparently having no concerns regarding antibiotic use in general, for self-limiting minor conditions such as colds and flu or non-infectious conditions such as headaches. A significant portion of senior pharmacy students reported that antibiotics can be used for urinary tract infections and skin wound infection rather than cold and flu illnesses, which are non-bacterial in aetiology, and thus unresponsive to antibiotics. Hence, these findings reiterate that senior pharmacy students have better knowledge of antibiotics compared to their junior counterparts.

A substantial proportion of the students had become familiar with many of the terms related to AMR as they progressed through their undergraduate pharmacy studies. Significantly more senior pharmacy students had knowledge of AMR-related terms. This study suggests that knowledge of these terms is associated with a greater knowledge of antibiotics generally and would likely lead to a reduction in the indiscriminate use of antibiotics.

### Meaning of the study: Possible mechanisms and implications for clinicians or policymakers

A high prevalence of antibiotic use among pharmacy student population in Sri Lanka was demonstrated in this study. The findings of this study suggest that entry-level (junior) pharmacy students hold some misconceptions about the appropriate use of antibiotics, knowledge on antibiotics and AMR. A comparison between junior and senior pharmacy students suggests that pharmacy education is associated with improved understanding of appropriate antibiotic use, enhanced the knowledge on antibiotics and AMR among undergraduate pharmacy students in Sri Lanka. There is limited information regarding antibiotic use and AMR understanding among pharmacy students from South Asia including Sri Lanka. To our knowledge, this is the first study undertaken as a nationwide survey to assess antibiotic and AMR knowledge among pharmacy undergraduate students in a South Asian country. The results of this study are important not only for Sri Lanka but also for all South Asia nations as recent findings have shown a low level of knowledge on AMR among health care professionals in developing countries [41].

The baseline data for this study suggests that socio-economic factors may influence the inappropriate use of antibiotics. In Sri Lanka, antibiotics are inexpensive and obtainable as non-prescription medicines from most of the pharmacies [42, 43]. Further, it was highlighted prior experience with antibiotics and time efficiency to consult physicians were main reasons behind adherence to self-medication practices of antibiotics [44]. Moreover, there are insufficient facilities for laboratory testing [45] and prescribing antibiotics without accompanying information from laboratory investigations is common [46, 47]. Hence, there are significant facilitators for Sri Lankans to inappropriately use antibiotics for minor ailments [48, 49]. Such behaviours have been reflected by the junior pharmacy students in this study, who have had limited exposure to education about antibiotics or AMR.

Therefore, it is advisable that government authorities establish a strict regulatory and legislative framework for antibiotic prescription, distribution and dispensing practices. Pharmacy personnel should dispense antibiotics only if an authentic prescription is presented to them and should promote practices whereby consumers will seek medical advice for conditions likely to be responsive to antibiotics and not use antibiotics inappropriately.

### Unanswered questions and future research

This study had been conducted among pharmacy students. A worthwhile extension of this research would be to replicate the study among practicing pharmacists, doctors, dentists and allied health professional and their respective students to investigate their knowledge, attitude and practice towards antibiotics. Further research in the general population regarding antibiotic procurement and use is also warranted. Such research should provide essential data that would inform decisions relating to restrictions on access to antibiotics and interventions that support appropriate prescribing, dispensing and promotional activities concerning antibiotic use in Sri Lanka.

## Conclusion

In conclusion, the findings of this study provide the first prevalence data on antibiotic use and knowledge of antibiotics and AMR among Sri Lankan pharmacy undergraduate students. The study results indicate that current pharmacy education has appropriately influenced quality use of antibiotics among pharmacy students in Sri Lanka. Nevertheless, gaps remain in students’ knowledge and a collaborative and strategic education plan for all Sri Lankan pharmacy undergraduates would likely provide greater impact on antibiotic knowledge and AMR among pharmacy undergraduate students in Sri Lanka. This would ultimately benefit Sri Lankan consumers and other health care professionals. Such a strategy may provide a template for other developing countries in South Asia similar to Sri Lanka.

## Additional files

Additional file 1: Annexure 1. Details of response rate from every universities. (DOCX 12 kb)

Additional file 2: Annexure 2. Questionnaire. (DOCX 35 kb)

Additional file 3: Annexure 3. Frequency and percentage of response for the questions related to antibiotic use. (DOCX 13 kb)

Additional file 4: Annexure 4. Frequency and percentage of response for the questions and statements related to antibiotic use. (DOCX 13 kb)

Additional file 5: Annexure 5. Response for the disease conditions and knowledge on antibiotics. (DOCX 13 kb)

Additional file 6: Annexure 6. Frequency of response for the terms related to antimicrobial resistance heard from different sources. (DOCX 12 kb)

Additional file 7: Annexure 7. Response for the use related to antibiotic use in agriculture and in food producing animals. (DOCX 12 kb)

## Abbreviations

AMR: Antimicrobial resistance
B.Pharm: Bachelor of Pharmacy
KDU: Kothalawala Defence University
OUSL: Open University of Sri Lanka
SPSS: Statistical Process for Social Sciences
UoJ: University of Jaffna
UoP: University of Peradeniya
UoR: University of Ruhuna
USJP: University of Sri Jayewardenepura
WHO: World Health Organization
