# Supplementary material for: Investigating knowledge regarding antibiotics and antimicrobial resistance among pharmacy students in Sri Lankan universities
Source: BMC Infect Dis. 2018 May 8;18:209. doi: 10.1186/s12879-018-3107-8 (PMC5941408; doi:10.1186/s12879-018-3107-8)
Supplement: Supplementary file 1 — Annexure 1. Details of response rate from every universities. (DOCX 12 kb) [file 12879_2018_3107_MOESM1_ESM.docx]

**Additional file 1: Annexure 1.** Details of response rate from every universities

| **Name of University** | **Number of Students Enrolled *** | **Number of students responded** | **Response rate (%)** |
| --- | --- | --- | --- |
| University of Peradeniya (UOP) | 114 | 102 | 90 |
| University of Sri Jayewardenepura (USJP) | 111 | 96 | 86 |
| University of Jaffna (UOJ) | 78 | 65 | 83 |
| University of Ruhuna (UOR) | 75 | 62 | 83 |
| Kotelawala Defence University (KDU) | 103 | 84 | 82 |
| Open University of Sri Lanka (OUSL) | 257 | 57 | 22 |
| *During data collection period |  |  |  |
