# Supplementary material for: Investigating knowledge regarding antibiotics and antimicrobial resistance among pharmacy students in Sri Lankan universities
Source: BMC Infect Dis. 2018 May 8;18:209. doi: 10.1186/s12879-018-3107-8 (PMC5941408; doi:10.1186/s12879-018-3107-8)
Supplement: Supplementary file 2 — Annexure 2. Questionnaire. (DOCX 35 kb) [file 12879_2018_3107_MOESM2_ESM.docx]

**Additional file 2: Annexure 2.**

QUESTIONNAIRE

DEMOGRAPHICS: **Circle your answer**

**S1**. What is your age?

1. 20 – 25

2. 26 - 34

3. 35- 44

4. 45+

**S2**. What is your gender?

1. Male

2. Female

**S3.** What is your name of the University?

1. University of Peradeniya

2. University of Sri Jayewardenepura

3. University of Jaffna

4. University of Ruhuna

5. Kothalawala Defence University (KDU)

6. Open University of Sri Lanka (OUSL)

**S4**. What is your year of study at university?

1. 1st year

2. 2nd year

3. 3rd year

4. 4th year

MAIN QUESTIONNAIRE

USE OF ANTIBIOTICS

**Circle your answer**

**1)** When did you last take antibiotics?

1. In the last month

2. In the last 6 months

3. In the last year

4. More than a year ago

5. Never

6. Can’t remember

***If ‘Never’ circle code 5, go straight to Question 5***

**2)** On that occasion, did you get the antibiotics from a doctor’s prescription

1. Yes

2. No

3. Can’t remember

**3)** On that occasion, did you get advice from a doctor, nurse or pharmacist on how to take them

1. Yes, I received advice on how to take them ( e.g. with food for 7 days)

2. No

3. Can’t remember

**4)** On that occasion, where did you get the antibiotics?

1. Medical store or Pharmacy

2. Stall or hawker

3. The internet

4. Friend or family member

5. I had them saved up from previous time

6. Somewhere/someone else

7. Can’t remember

KNOWLEDGE ABOUT ANTIBIOTICS

**5)** When do you think you should stop taking antibiotics once you’ve begun treatment?

1. When you feel better

2. When you’ve taken all of the antibiotics as directed

3. Don’t know

**6)** Do you think this statement is ‘true’ or ‘false?’

“It’s okay to use antibiotics that were given to a friend or family member, as long as they were used to treat the same illness”

1. True

2. False

3. Don’t know

**7)** Do you think this statement is ‘true’ or ‘false?’

“It’s okay to buy the same antibiotics, or request these from a doctor if you are sick and they helped you get better when you had the same symptoms before”

1. True

2. False

3. Don’t know

**8)** Do you think these conditions can be treated with antibiotics?

You may circle more than one answer

1. HIV/AIDS

2. Gonorrhoea

3. Bladder infection or urinary tract infection (UTI)

4. Diarrhoea

5. Cold and flu

6. Fever

7. Malaria

8. Measles

9. Skin or wound infection

10. Sore throat

11. Body aches

12. Headaches

KNOWLEDGE ON ANTIBIOTIC RESISTANCE

**9)** Have you heard any of the following terms? You may circle more than one answer

1. Antibiotic resistance

2. Superbugs

3. Antimicrobial resistance

4. AMR

5. Drug resistance

6. Antibiotic resistant bacteria

**10)** You may circle more than one answer for 10.1, 10.2, 10.3, 10.4, 10.5 and 10.6

**10.1.** If answered Yes in Question 9) to ‘Antibiotic resistance’. Where did you hear about the term: ‘Antibiotic Resistance’?

1. Doctor or nurse

2. Pharmacist

3. Family member or friend (including on social media

4. Media (newspaper, TV, radio)

5. Specific campaign

6. Undergraduate study

7. Can’t remember

**10.2**. If answered Yes in Question 9) to ‘Superbugs’

Where did you hear about the term: ‘Superbugs’?

1. Doctor or nurse

2. Pharmacist

3. Family member or friend (including social media)

4. Media (newspaper, TV, radio)

|  | 5. | Specific campaign |
| --- | --- | --- |
|  | 6. | Undergraduate study |
|  | 7. | Can’t remember |
| **10.3.** |  | If answered Yes in Question 9) to ‘Antimicrobial resistance’  Where did you hear about the term: ‘Antimicrobial resistance’? |
|  | 1. | Doctor or nurse |
|  | 2. | Pharmacist |
|  | 3. | Family member or friend (including social media) |
|  | 4. | Media (newspaper, TV, radio) |
|  | 5. | Specific campaign |
|  | 6. | Undergraduate study |
|  | 7. | Can’t remember |
| **10.4**. |  | If answered Yes in Question 9) to ‘AMR’  Where did you hear about the term: ‘AMR’ |
|  | 1. | Doctor or nurse |
|  | 2. | Pharmacist |
|  | 3. | Family member or friend (including social media) |
|  | 4. | Media (newspaper, TV, radio) |
|  | 5. | Specific campaign |
|  | 6. | Undergraduate study |
|  | 7. | Can’t remember |
| **10.5**. |  | If answered Yes in Question 9) to ‘Drug resistance’  Where did you hear about the term: ‘Drug Resistance?’ |
|  | 1. | Doctor or nurse |
|  | 2. | Pharmacist |
|  | 3. | Family member or friend (including social media) |
|  | 4. | Media (newspaper, TV, radio) |
|  | 5. | Specific campaign |
|  | 6. | Undergraduate study |
|  | 7. | Can’t remember |
| **10.6**. |  | If answered Yes in Question 9) to ‘Antibiotic- resistant bacteria’ |
|  |  | Where did you hear the term: ‘Antibiotic resistant bacteria’? |
|  | 1. | Doctor or nurse |
|  | 2. | Pharmacist |
|  | 3. | Family member or friend (including social media) |
|  | 4. | Media (newspaper, TV, radio) |
|  | 5. | Specific campaign |
|  | 6. | Undergraduate study |
|  | 7. | Can’t remember |

**11)** Please indicate whether you think the following statements are ‘true’ or ‘false’

|  | Statement | **True** | **False** | **Do not Know** |
| --- | --- | --- | --- | --- |
| 1 | Antibiotic resistance occurs when your body becomes resistant to  antibiotics and they no longer works as well |  |  |  |
| 2 | Many infections are becoming increasingly resistant to treatment by  antibiotics |  |  |  |
| 3 | If bacteria are resistant to antibiotics, it can be very difficult or  impossible to treat the infections they cause |  |  |  |
| 4 | Antibiotic resistance is an issue that could affect me or my family |  |  |  |
| 5 | Antibiotic resistance is an issue in other countries but not here |  |  |  |
| 6 | Antibiotic resistance is only a problem for people who take antibiotics  regularly |  |  |  |
| 7 | Bacteria which are resistant to antibiotics can be spread from person to person  person |  |  |  |
| 8 | Antibiotic-resistant infections could make medical procedures like  surgery, organ transplants and cancer treatment much more difficult ddidifficultdangerous |  |  |  |

**12)** On the scale down shown, how much do you agree the following actions would help address the problem of antibiotic resistance?

|  | Statement | **Agree**  **Strongly** | **Agree**  **Slightly** | **Neither agree nor**  **disagree** | **Disagree**  **Slightly** | **Disagree**  **Strongly** |
| --- | --- | --- | --- | --- | --- | --- |
| 1 | People should use antibiotics only  when they are prescribed by a doctor |  |  |  |  |  |
| 2 | Farmers should give fewer antibiotics  to food-producing animals |  |  |  |  |  |
| 3 | People should not keep and use them  later for other illnesses |  |  |  |  |  |
| 4 | Parents should make sure all of their  children’s vaccinations are up to date |  |  |  |  |  |
| 5 | People should wash their hands  regularly |  |  |  |  |  |
| 6 | Doctors should only prescribe  antibiotics when they are needed |  |  |  |  |  |
| 7 | Governments should reward the  development of new antibiotics |  |  |  |  |  |
| 8 | Pharmaceutical companies should  develop new antibiotics |  |  |  |  |  |

**13)** On the scale shown, how much do you agree with following statement?

|  | Statement | **Agree**  **Strongly** | **Agree**  **Slightly** | **Neither**  **agree nor disagree** | **Disagree**  **Slightly** | **Disagree**  **Strongly** |
| --- | --- | --- | --- | --- | --- | --- |
| 1 | Antibiotic resistance is one of the  biggest problems the world faces |  |  |  |  |  |
| 2 | Medical experts will solve the problem  of antibiotic resistance before it becomes too serious |  |  |  |  |  |
| 3 | Everyone needs to take responsibility  for using antibiotics responsibly |  |  |  |  |  |
| 4 | There is not much people like me can  do to stop antibiotic resistance |  |  |  |  |  |
| 5 | I am worried about the impact that  antibiotic resistance will have on my health, and that of my family |  |  |  |  |  |
| 6 | I am not at risk of getting an antibiotic  resistant infection, as long as I take my antibiotic correctly |  |  |  |  |  |

USE OF ANTIBIOTICS IN AGRICULTURE

**14)** Do you think antibiotics are widely used in agriculture, including food producing animals in your country?

1. Yes

2. No

3. Don’t know
