# Supplementary material for: Investigating knowledge regarding antibiotics and antimicrobial resistance among pharmacy students in Sri Lankan universities
Source: BMC Infect Dis. 2018 May 8;18:209. doi: 10.1186/s12879-018-3107-8 (PMC5941408; doi:10.1186/s12879-018-3107-8)
Supplement: Supplementary file 3 — Annexure 3. Frequency and percentage of response for the questions related to antibiotic use. (DOCX 13 kb) [file 12879_2018_3107_MOESM3_ESM.docx]

**Additional file 3: Annexure 3.** Frequency and percentage of response for the questions related to antibiotic use

| **Questions** | **Junior Students (n=260)** | **Senior Students (n=206)** |
| --- | --- | --- |
|  | Frequency (%) | Frequency (%) |
| When did you last take antibiotics? |  |  |
| In the last month | 83 (32) | 49 (24) |
| In the last 6 months | 80 (31) | 90 (44) |
| In the last year | 24 (9) | 26 (13) |
| More than a year ago | 25 (10) | 26 (13) |
| Never | 2 (1) | 0 (0) |
| Can't remember | 44 (17) | 15 (7) |
|  |  |  |
| Did you get the antibiotics from a doctor’s prescription? |  |  |
| Yes | 200 (79) | 159 (78) |
| No | 47 (19) | 40 (20) |
| Can't remember | 7 (3) | 5 (2) |
|  |  |  |
| Did you get advice from a doctor, nurse or pharmacist on how to take them? |  |  |
| Yes | 175 (69) | 92 (45) |
| No | 64 (25) | 101 (50) |
| Can't remember | 15 (6) | 11 (5) |
| On that occasion, where did you get the antibiotics? |  |  |
| Pharmacy | 230 (92) | 190 (93) |
| Stall / hawker | 1 (0) | 0 (0) |
| The internet | 0 (0) | 0 (0) |
| Friend / family member | 6 (2) | 4 (2) |
| Saved from previous experience | 6 (2) | 4 (2) |
| Somewhere / Someone | 2 (1) | 2 (1) |
| Can't remember | 5 (2) | 4 (2) |
