# Supplementary material for: Investigating knowledge regarding antibiotics and antimicrobial resistance among pharmacy students in Sri Lankan universities
Source: BMC Infect Dis. 2018 May 8;18:209. doi: 10.1186/s12879-018-3107-8 (PMC5941408; doi:10.1186/s12879-018-3107-8)
Supplement: Supplementary file 4 — Annexure 4. Frequency and percentage of response for the questions and statements related to antibiotic use. (DOCX 13 kb) [file 12879_2018_3107_MOESM4_ESM.docx]

**Additional file 4: Annexure 4.** Frequency and percentage of response for the questions and statements related to antibiotic use

| **Question / Statements** | **Junior Students (n=260)** | **Senior Students (n=206)** |
| --- | --- | --- |
|  | Frequency (%) | Frequency (%) |
| When do you think you should stop taking antibiotics once you’ve begun treatment? |  |  |
| When you feel better | 78 (30) | 21 (10) |
| When you have taken all | 154 (60) | 180 (87) |
| Don't know | 25 (10) | 5 (2 ) |
| “It’s okay to use antibiotics that were given to a friend |  |  |
| or family member, as long as they were used to treat the same illness” |  |  |
| True | 9 (4) | 4 (2) |
| False | 241 (94) | 200 (97) |
| Don't Know | 7 (3) | 2 (1) |
|  |  |  |
| “It’s okay to buy the same antibiotics, or request these from a doctor if you are sick |  |  |
| and they helped you get better when you had the same symptoms before” |  |  |
| True | 24 (9) | 19 (9) |
| False | 202 (79) | 180 (87) |
| Don't Know | 29 (11) | 7 (3) |
