# Supplementary material for: Investigating knowledge regarding antibiotics and antimicrobial resistance among pharmacy students in Sri Lankan universities
Source: BMC Infect Dis. 2018 May 8;18:209. doi: 10.1186/s12879-018-3107-8 (PMC5941408; doi:10.1186/s12879-018-3107-8)
Supplement: Supplementary file 5 — Annexure 5. Response for the disease conditions and knowledge on antibiotics. (DOCX 13 kb) [file 12879_2018_3107_MOESM5_ESM.docx]

| **Additional file 5: Annexure 5.** Response for the disease conditions and knowledge on antibiotics   \|  \| **Junior** (n=260) \| **Senior** (n=206) \| *** *P* value*** \| \| --- \| --- \| --- \| --- \| \|  \| Frequency (%) \| Frequency (%) \|  \| \| HIV \| 18 (7) \| 34 (17) \| .001 \| \| Gonorrhoea \| 71 (27) \| 68 (33) \| .000 \| \| Bladder infection \| 166 (64) \| 187 (91) \| .000 \| \| Diarrhoea \| 115 (44) \| 112 (54) \| .030 \| \| Cold / Flu \| 147 (57) \| 90 (44) \| .006 \| \| Fever \| 119 (46) \| 72 (35) \| .018 \| \| Malaria \| 68 (26) \| 36 (17) \| .025 \| \| Measles \| 27 (10) \| 15 (7) \| .245 \| \| Skin wound infection \| 194 (75) \| 188 (91) \| .000 \| \| Sore throat \| 134 (52) \| 133 (65) \| .005 \| \| Body aches \| 30 (12) \| 20 (10) \| .526 \| \| Headaches \| 21 (8) \| 6 (3) \| .018 \|   ****P*** value: P<0.05 considered significant |  |  |  |  |
| --- | --- | --- | --- | --- | --- | --- | --- | --- | --- | --- | --- | --- | --- | --- | --- | --- | --- | --- | --- | --- | --- | --- | --- | --- | --- | --- | --- | --- | --- | --- | --- | --- | --- | --- | --- | --- | --- | --- | --- | --- | --- | --- | --- | --- | --- | --- | --- | --- | --- | --- | --- | --- | --- | --- | --- | --- | --- | --- | --- | --- |
