# Supplementary material for: Investigating knowledge regarding antibiotics and antimicrobial resistance among pharmacy students in Sri Lankan universities
Source: BMC Infect Dis. 2018 May 8;18:209. doi: 10.1186/s12879-018-3107-8 (PMC5941408; doi:10.1186/s12879-018-3107-8)
Supplement: Supplementary file 6 — Annexure 6. Frequency of response for the terms related to antimicrobial resistance heard from different sources. (DOCX 12 kb) [file 12879_2018_3107_MOESM6_ESM.docx]

**Additional file 6: Annexure 6.** Frequency of response for the terms related to antimicrobial resistance heard from different sources

| **Terms related to AMR** | **Doctor / Nurse** | **Pharmacist** | **Family member / friend** | **Media** | **Specific campaign** | **Undergraduate education** |
| --- | --- | --- | --- | --- | --- | --- |
| Antibiotic Resistance | 70 | 93 | 72 | 125 | 40 | 358 |
| Superbugs | 3 | 5 | 5 | 14 | 2 | 26 |
| Antimicrobial Resistance | 37 | 56 | 41 | 67 | 22 | 311 |
| AMR | 4 | 6 | 7 | 14 | 1 | 48 |
| Drug Resistance | 65 | 71 | 57 | 86 | 31 | 305 |
| Antibiotic Resistant bacteria | 36 | 47 | 54 | 73 | 27 | 343 |
