# Supplementary material for: Investigating knowledge regarding antibiotics and antimicrobial resistance among pharmacy students in Sri Lankan universities
Source: BMC Infect Dis. 2018 May 8;18:209. doi: 10.1186/s12879-018-3107-8 (PMC5941408; doi:10.1186/s12879-018-3107-8)
Supplement: Supplementary file 7 — Annexure 7. Response for the use related to antibiotic use in agriculture and in food producing animals. (DOCX 12 kb) [file 12879_2018_3107_MOESM7_ESM.docx]

**Additional file 7: Annexure 7.** Response for the use related to antibiotic use in agriculture and in food producing animals

| **Statement** | **Junior (n=260)** | **Senior (n=206)** |
| --- | --- | --- |
|  | Frequency (%) | Frequency (%) |
| Do you think antibiotics are widely used in agriculture, including food producing animals in your country? |  |  |
| Yes | 99 (39) | 76 (38) |
| No | 52 (21) | 25 (13) |
| Don't know | 102 (40) | 99 (50) |
